# Supplementary material for: Improved Dipole Source Localization from Simultaneous MEG-EEG Data by Combining a Global Optimization Algorithm with a Local Parameter Search: A Brain Phantom Study
Source: Bioengineering (Basel). 2024 Sep 6;11(9):897. doi: 10.3390/bioengineering11090897 (PMC11428344; doi:10.3390/bioengineering11090897)
Supplement: Supplementary file 1 [file bioengineering-11-00897-s001.zip › bioengineering-3188039-supplementary.pdf]

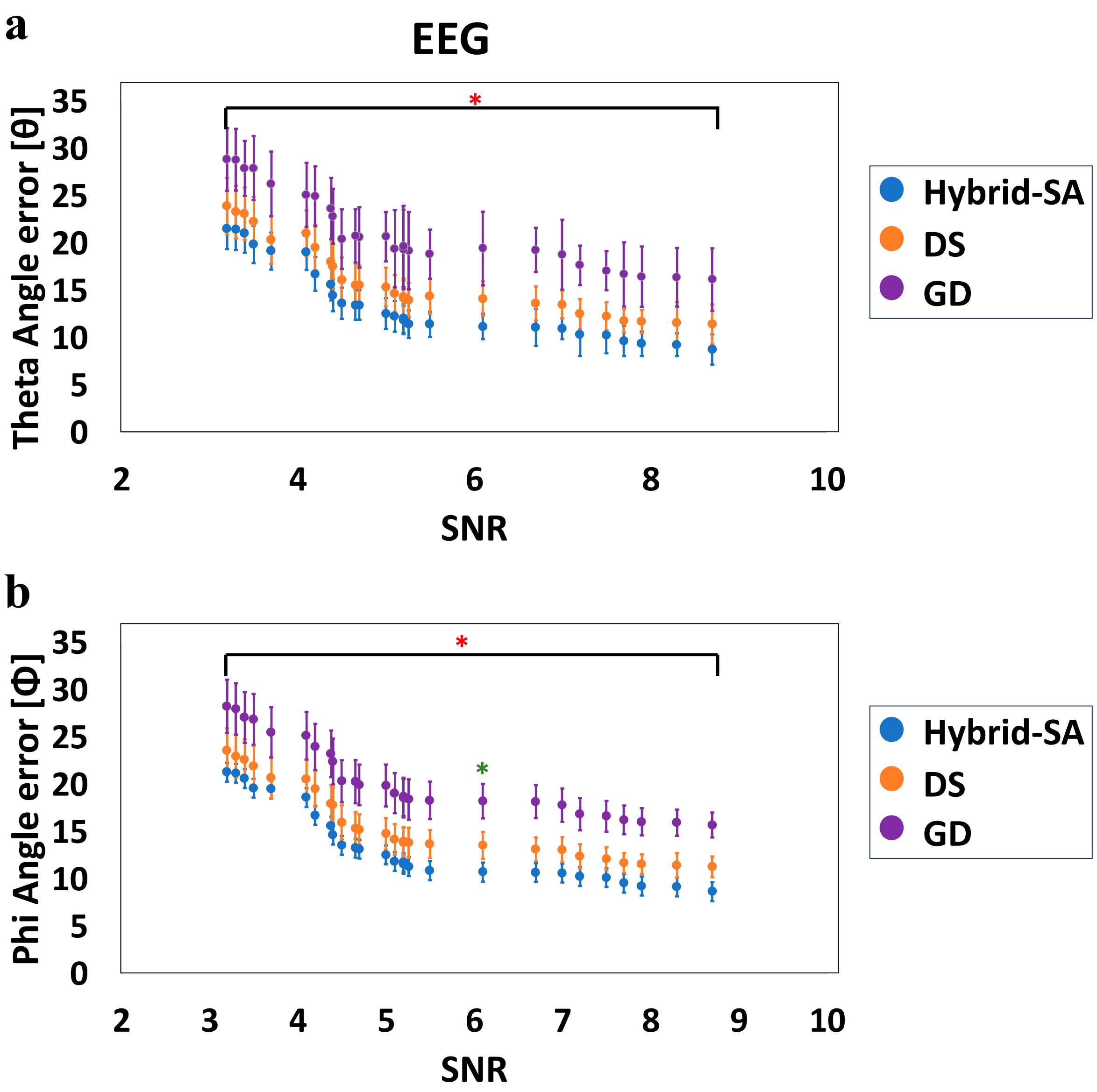

Figure S1. Comparison between the three algorithms, Hybrid-SA, DS, and GD for (a) dipole theta (polar) angle error, and (b) dipole phi (azimuthal) angle error in degrees versus SNR for EEG. (* representing statistically significant difference between Hybrid-SA and GD, * representing statistically significant difference between Hybrid-SA and DS, for $p < 0.05$).

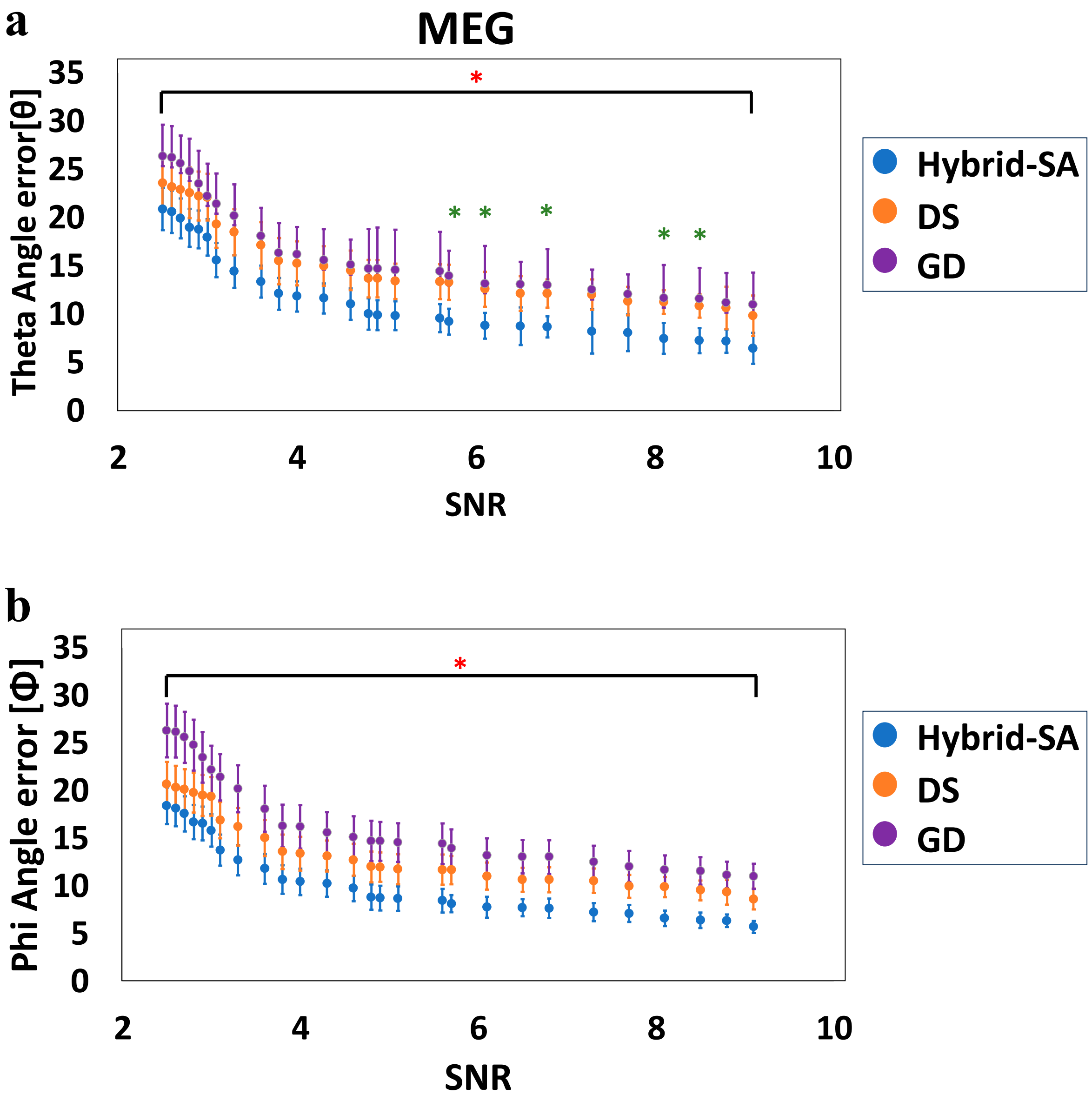

Figure S2. Comparison between the three algorithms, Hybrid-SA, DS, and GD for (a) dipole theta (polar) angle error, and (b) dipole phi (azimuthal) angle error in degrees versus SNR for MEG. (* representing statistically significant difference between Hybrid-SA and GD, * representing statistically significant difference between Hybrid-SA and DS, for $p < 0.05$).
